# Assessment of the RNA Silencing Suppressor Activity of Protein P0 of Pepper Vein Yellows Virus 5: Uncovering Natural Variability, Relevant Motifs and Underlying Mechanism

**DOI:** 10.3390/biology11121801

**Published:** 2022-12-12

**Authors:** Miryam Pérez-Cañamás, Mónica Bustos, Victoria Puértolas, Yolanda Castelló, Sofía Peiró, Carmen Hernández

**Affiliations:** Instituto de Biología Molecular y Celular de Plantas (Consejo Superior de Investigaciones Científicas-Universidad Politécnica de Valencia), Ciudad Politécnica de la Innovación, Calle Ingeniero Fausto Elio, Ed. 8E, Camino de Vera s/n, 46022 Valencia, Spain

**Keywords:** *Pepper vein yellows virus* 5, polerovirus, viral supressor of RNA silencing, protein P0, AGO degradation

## Abstract

**Simple Summary:**

Viruses must face their host’s antiviral defenses during the infection process. In plants, one of the main antiviral mechanisms is based on RNA silencing, which ultimately leads to the degradation of viral RNA. To cope with this host’s strategy, plant viruses encode proteins that act as suppressors of RNA silencing (VSRs). These proteins are structurally diverse and may differ in their mode of action. Due to their essential role for viral fitness, VSRs are appealing targets for virus control. In this study, we provide significant information on the structural and functional properties of the VSR encoded by *Pepper vein yellows virus* 5, a pathogen belonging to a group of emergent and closely related poleroviruses that threaten pepper cultivation worldwide. This virus caused an outbreak nine years ago in Spain and we also present data showing that the virus is still present in the country.

**Abstract:**

*Pepper vein yellows virus* 5 (PeVYV-5) belongs to a group of emerging poleroviruses (family *Solemoviridae*) which pose a risk to pepper cultivation worldwide. Since its first detection in Spain in 2013 and the determination of the complete genome sequence of an isolate in 2018, little is known on the presence, genomic variation and molecular properties of this pathogen. As other members of genus *Polerovirus*, PeVYV-5 encodes a P0 protein that was predicted to act as viral suppressor of RNA silencing (VSR), one of the major antiviral defense mechanisms in plants. The results of the present work have indicated that PeVYV-5 P0 is a potent VSR, which is able to induce the degradation of Argonaute (AGO) endonucleases, the main effectors of RNA silencing. New viral isolates have been identified in samples collected in 2020–2021 and sequencing of their P0 gene has revealed limited heterogeneity, suggesting that the protein is under negative selection. Analysis of natural and engineered P0 variants has pinpointed distinct protein motifs as critical for the VSR role. Moreover, a positive correlation between the VSR activity of the protein and its capability to promote AGO degradation could be established, supporting that such activity essentially relies on the clearance of core components of the RNA silencing machinery.

## 1. Introduction

Viruses must cope with the antiviral defenses of the host in order to survive. In plants, one of the main antiviral responses is based on RNA silencing [1]. This response is triggered by viral double-stranded (ds) RNA, or highly structured single-stranded (ss) RNA molecules that are recognized by Dicer-like (DCL) type III RNases to generate small RNAs (sRNAs) of 21–24 nt. A strand of these small duplexes is incorporated to an RNA-induced silencing complex (RISC) whose main component is an endonuclease of the Argonaute (AGO) family. The RISC-loaded sRNA will guide the complex to cognate RNAs to induce their AGO-mediated degradation. The silencing process is amplified in plants through the action of cellular RNA-dependent RNA polymerases (RDR) that may use products resulting from AGO slicing, or other aberrant RNAs, to produce new dsRNAs that will act as DCL substrates, thus giving rise to secondary sRNAs. This amplification seems essential to produce mobile signals (that include sRNAs of 22 nt) that spread silencing from the site it was firstly initiated to distal parts of the plant [2].

To counteract antiviral silencing, plant viruses usually encode proteins known as viral suppressors of RNA silencing (VSR) that may interfere with the silencing process at distinct stages [3,4]. One of the most common mechanisms of action of VSRs consists in sRNA binding, which precludes sRNA incorporation into RISC. Protein p19 encoded by tombusviruses is a paradigmatic and well-studied case of this type of strategy [5,6], though many other VSRs have been reported with the same mode of action [7,8,9,10]. Some VSRs are able to associate to dsRNAs in a size-independent manner, sequestering both DCL substrates and RISC cargoes. Examples of VSRs following this trick are protein p38, encoded by *Turnip crinkle virus*, or the aureusviral p14 [11,12]. Another relatively common VSR strategy is based on interference with protein components of the silencing pathway, particularly AGOs, which are the core effectors of RISC. Though interactions of VSRs with AGO proteins have been frequently detected, the functional implications of those interactions are not always well understood [13]. Nevertheless, evidence that those interactions may prevent RISC assembly, RISC activity or affect the AGO stability has been obtained in distinct cases (e.g., [14,15,16]). 

Protein P0 is a polypeptide encoded by members of genus *Polerovirus* (26 recognized species) and *Enamovirus* (5 recognized species) within family *Solemoviridae*. These viruses have a monopartite ssRNA genome of plus polarity of about 5,5–6 kb which harbors 5–8 genes, being the 5′-proximal gene the one that encodes P0 [17]. The activity as VSR of protein P0 was demonstrated for several viral species of these genera, though it could not be proven in other cases [18]. It should be noted in this context that the distinct P0 products share relatively low sequence similarity, with an average identity value of about 38 % [19]. Despite this, all of them seem to contain an F-box-like motif, of uncertain biological meaning, near the N-terminus, and several P0 proteins exhibit a conserved sequence, (K/R)IYGEDGX_3_FWR, at the C-terminus. The molecular basis of VSR activity has been analyzed for a few P0 proteins and the results supported that such activity relies on the promotion of AGO degradation [20,21]. Such effect was mainly tested on AGO1, one of the key slicers involved in antiviral defense in plants [13,22], though some assays indicated that it could be extensive to other AGO proteins [20,23]. The name “Pepper vein yellows virus” (PeVYV) has been used to designate a series of emerging and closely related poleroviruses (so far, at least ten) that have been noticed in distinct parts of the world and that threat pepper crops [24,25,26,27,28,29]. In 2013, one of these viruses, PeVYV-5, was detected in Spain and the genomic sequence of a viral isolate was later determined [24,30]. As other members of genus *Polerovirus*, PeVYV-5 potentially encodes two proteins involved in replication (P1 and its frameshift product P1/P2, the viral RDR), two movement proteins (P3a and P4) and two capsid proteins (P3 and its read-through product P3/P5). In addition, the 5′-proximal gene encodes a P0 protein whose function as VSR is presumed but not yet corroborated. Indeed, such a function has only been demonstrated so far for one PeVYV species, PeVYV-3, which was reported in China [31,32]. 

Here we aimed to assess the potential VSR activity of PeVYV-5 P0 and, if so, to explore molecular traits underlying such activity. The results have revealed that PeVYV-5 P0 is a strong VSR, which is able to promote efficient degradation of AGO1 as well as of other AGOs. Identification of new PeVYV-5 isolates allowed us to explore natural P0 sequence polymorphism. Analysis of natural and artificially engineered P0 variants pinpointed relevant motifs for VSR activity. Moreover, a correlation between such activity and the capability of the protein to induce AGO decay could be established, further supporting that this mechanism is shared by distinct P0 proteins irrespective of their sequence particularities. 

## 2. Materials and Methods

### 2.1. Viral Isolates and RT-PCR Amplification

Six pepper plants with PeVYV symptoms and tested positive for the virus with primers designed for diagnosis (PY-TV-F2972 and PY-TV-R3531 in [33]), were used to amplify the corresponding P0 gene. These plants were collected from greenhouses located in Balanegra (isolates BN1-4) and Vicar (isolates VC1-2), two villages from Almería (Spain), in 2020 and 2021, respectively. Total RNA was obtained from these plants (see below) and employed for RT-PCR amplification of the corresponding P0 gene with SuperScript IV One-Step RT-PCR System (Thermo Fisher Scientific, Waltham, Massachusetts) and virus specific primers (Table 1). The DNA products were run in 1% agarose gels, purified with GeneJet Gel Extraction kit (Thermo Fisher Scientific, Waltham, Massachusetts) and sequenced with specific oligonucleotides. In addition, as mentioned in the next section, the P0 gene from one isolate identified in Almería in 2013 (isolate ES-Alm2-1, called hereafter reference isolate), when an important viral outbreak was reported [24,30], was also employed in distinct parts of the present study. 

### 2.2. DNA Constructs

The P0 gene from the PeVYV-5 reference isolate, which was sequenced in [24], was PCR-amplified from a proper plasmid using specific primer pairs with suitable restriction sites at their 5′end and the KAPA HiFi HotStart PCR kit (Kapa Biosystems). In addition, the P0 gene from isolate BN2 of PeVYV-5 was also selected for further cloning and analysis. Untagged P0 or the P0 genes with a sequence encoding a composed HAFlag tag at the 3′-termini, were produced and inserted between the *Cauliflower mosaic virus* (CaMV) 35S promoter and the terminator sequence of the *Solanum tuberosum* proteinase inhibitor II gene (PoPit), and cloned into the binary vector pMOG800 [34].

Specific mutations were introduced into the cloned HAFlag-tagged P0 gene of the reference isolate (P0^REF^) by PCR with the QuickChange site-directed mutagenesis kit (Stratagene) and appropriate oligonucleotides. Five different protein mutants were generated by modifying two segments particularly rich in basic amino acids located between positions 17–22 (mutant 1) and 77–81 (mutant 4), respectively, the F-box motif (mutants 2 and 3) and a segment embracing positions 210–214 at the C-terminus (mutant 5). The last segment bears some of the amino acids of the motif (K/R)IYGEDGX_3_FWR which is conserved in a series of P0 proteins, though it is not strictly present in PeVYV-5 P0. Binary constructs allowing expression of GFP and p37, the VSR encoded by *Pelargonium line pattern virus* (PLPV), have been described elsewhere [9]. Binary plasmids for expression of AGO proteins from *Arabidopsis thaliana* tagged at their N-terminus with three tandem repeats of the hemagglutinin (HA) epitope (AGO1, AGO2) or with a single HA epitope (AGO3, AGO4, AGO5) were reported previously [35,36].

All newly generated constructs were routinely sequenced with an ABI PRISM DNA sequencer ABI 3130 XL (PerkinElmer Life Sciences) to avoid unwanted modifications. The primers used to generate the distinct recombinant plasmids are listed in Table 1.

### 2.3. Sequence Analyses 

Multiple sequence alignments were constructed using Clustal Omega [37]. The translation of nucleotide sequences into amino acid sequences was performed with the aid of the Translate tool at the Expasy Portal (https://www.expasy.org/ (accessed on 14 June 2021). Pairwise sequence comparisons were performed with BLASTn /BLASTp to calculate sequence identities. The intensity of selection, described as the ratio between non-synonymous (d_N_) and synonymous (d_S_) substitution rates, was analyzed using some methods available in the HYPHY package [38] in the www.datamonkey.org (accessed on 5 October 2022) server.

The sequences reported in this paper have been deposited in the GenBank under accession numbers OP778220-OP778225.

### 2.4. Agroinfiltration Assays and Fluorescence Imaging

All binary plasmid constructs were transformed into *Agrobacterium tumefaciens* strain C58C1 by freeze–thaw shock method. Cultures of *A. tumefaciens* transformed with each plasmid were sedimented, resuspended in agroinfiltration buffer (MES 10 mM, MgCl_2,_ acetosyringone 100 mM) at an OD_660_ of 0.5, incubated for 2 h and then used to infiltrate the abaxial side of two half-expanded leaves from 3-week-old *Nicotiana benthamiana* plants. In co-infiltration assays, equal volumes of the corresponding bacterial cultures (OD_660_ = 0.5) were mixed before infiltration. Mock infiltrated plants were used as controls. Two leaves per plant were infiltrated (with 2.5 mL of the infiltration mixture each) and at least three plants were employed in every case. A minimum of three replicates per experiment was done. Infiltrated plants were kept under greenhouse conditions (16 h days at 24 °C, 8 h nights at 20 °C) and leaves were harvested at distinct times post-infiltration for further analysis. 

When applicable, a visual detection of GFP fluorescence in agroinfiltrated leaf patches was performed using a fluorescence stereomicroscope (MZ16F Leica). Pictures were taken with a digital camera DFC300 FX Leica. 

### 2.5. RNA Extraction and Northern Blot Analysis

Total RNA was extracted from plant tissue (a mixture of three agroinfiltrated leaves) with extraction buffer (350 mM glycine, 48 mM NaOH, 340 mM NaCl, 34 mM EDTA, 4 % SDS) plus saturated phenol (2 mL per gram of plant material), and then fractionated with 2 M LiCl [39]. The RNA fractions insoluble and soluble in 2 M LiCl were subjected to Northern blot analysis for GFP mRNA and GFP-specific sRNAs detection, respectively, as indicated previously [9]. In both cases, a radioactive RNA probe synthesized *in vitro* from the GFP gene (minus polarity) was employed. Hybridization signals were visualized by autoradiography. For RT-PCR amplification of P0 gene from viral isolates, the RNA fractions insoluble in 2 M LiCl obtained from infected pepper samples were employed. 

### 2.6. Protein Extraction and Western Blot Analysis

The agroinfiltrated leaf material (a mixture of three agroinfiltrated leaves) was grinded with liquid nitrogen and the resulting leaf powder was thoroughly mixed with a protein extraction buffer (100 mM KCl, 0.1% NP40, 50 mM Tris-HCl pH 7.5, 2.5 mM MgCl_2_, 1 µg/mL leuptin, 0.5 mM PMSF, 1 µg/mL tripsin) supplemented with complete protease inhibitor cocktail (Roche Diagnostics GmbH), at a rate of 4 mL per gram of plant tissue. After centrifugation of the pellet cellular debris, the supernatants were collected and subjected to a Western blot analysis. To this aim, aliquots of the obtained protein extracts were resolved in SDS-PAGE, transferred to polyvinylidene difluoride (PVDF) membranes (Roche Diagnostics GmbH) and immunoblotted with an HRP-conjugated anti-HA antibody (3F10; Roche Diagnostics GmbH). Immunoreactive bands were revealed with Western Blot ultra sensitive HPR substrate following supplier´s recommendations (Takara Bio). Signals were recorded and quantified with the aid of an Amersham ImageQuant™ 800 GxP biomolecular imager (Cytiva).

## 3. Results

### 3.1. PeVYV-5 P0 Acts as Efficient VSR

**Figure 1:**
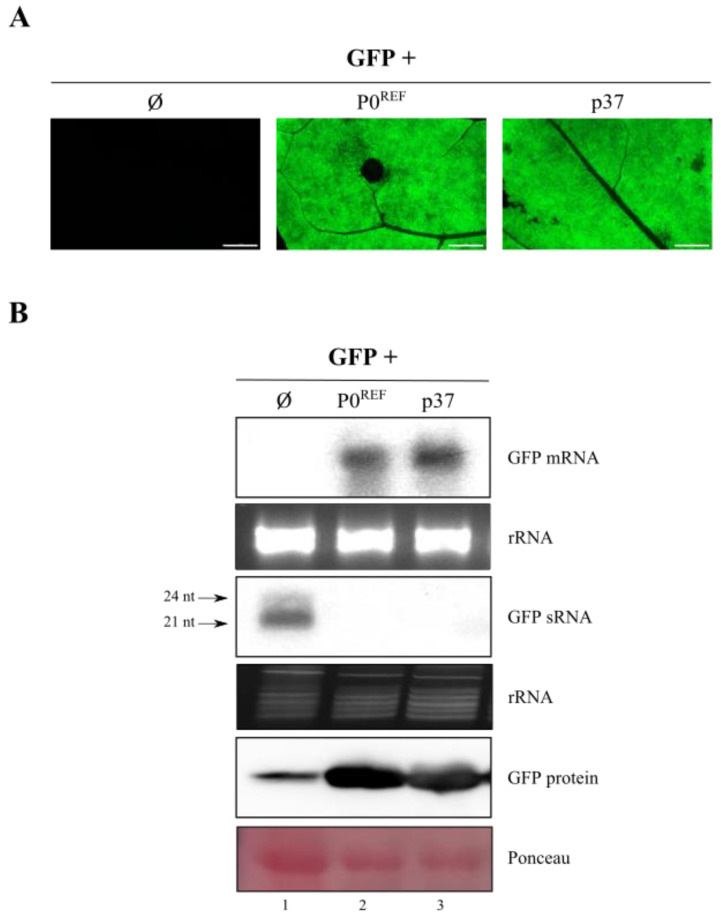
Assessment of RNA silencing suppressor activity of PeVYV-5 P0. *N. benthamiana* leaves were agroinfiltrated with constructs for expression of GFP either alone (Ø) or in combination with constructs directing expression of PeVYV-5 P0^REF^ or PLPV p37. (**A**) GFP fluorescence at 10 dpif in infiltrated leaf patches. The inset scale bar corresponds to 5 mm in all panels. (**B**) Northern blot hybridization and Western blot analysis for detection of the GFP mRNA (upper panel), derived sRNAs (middle panel) or GFP protein (lower panel) in infiltrated tissues harvested at 10 dpif. Ethidium bromide staining of RNA is shown as loading control below Northern blot panels. Ponceau S staining is shown below Western results as loading control.

To assess the potential VSR activity of PeVYV-5 P0, we used a classical silencing suppression assay consisting of the infiltration of *N. benthamiana* leaves with *A. tumefaciens* containing a plasmid with the reporter GFP gene controlled by the CaMV 35S promoter. This approach leads to the transcription of GFP mRNA at high levels followed by the induction of RNA silencing at several days post-infiltration. The co-infiltration of this *A. tumefaciens* strain together with another harboring a plasmid encoding a VSR, blocks the onset of RNA silencing and the GFP is easily detected under UV light [40,41]. To follow this approach, the P0 gene of the reference isolate of PeVYV-5 [24], P0^REF^, was inserted into a binary vector under the control of the CaMV 35S promoter and the resulting clone was used to transform *A. tumefaciens.* For simplicity, hereafter we will refer below to *A. tumefaciens* strains containing a binary plasmid by the name of the plasmid they carried. Leaves expressing just GFP showed clear fluorescence at 3–5 days post-infiltration (dpif) which was almost completely silenced at 8 dpif and beyond, as determined by observations with a fluorescence stereomicroscope (Supplementary Figure S1). However, co-expression of P0 with GFP maintained a very bright green fluorescence which was comparable in intensity and persistence (at least 15 days) to that visualized in parallel assays with protein p37 encoded by PLPV (Figure 1A), and used as positive control of suppressor activity [9].

To corroborate the visual observations at the molecular level, a Northern blot analysis of the RNA extracted from infiltrated leaves was carried out employing a GFP probe. For this procedure, tissue was collected at 10 dpif, the time point at which differences in fluorescence intensities were clear (Figure 1A) and the leaves were still in a reasonably good condition. GFP mRNA accumulation was virtually undetectable in leaves infiltrated GFP construct alone whereas GFP mRNA accumulated at considerable levels in leaves co-expressing GFP and P0^REF^, similar to what was observed in leaves co-expressing GFP and p37 (Figure 1B, upper panel). As expected from a silencing process, high levels of GFP sRNAs were detected at 10 dpif in leaves infiltrated just with the GFP construct. In contrast, GFP sRNAs were hardly detected in leaves co-infiltrated with GFP plus P0^REF^ or with GFP plus p37 constructs (Figure 1B, middle panel). Consistent with the fluorescence and GFP mRNA levels, GFP showed very low accumulation in leaf material agroinfiltrated with the GFP construct whereas high GFP amounts were present in tissue agroinfiltrated with GFP plus P0^REF^ or p37 constructs (Figure 1B, lower panel, and Supplementary Figure S2). Collectively, the results supported that PeVYV-5 P0 is able to inhibit RNA silencing to an extent similar to that of p37, a potent VSR. 

### 3.2. PeVYV-5 P0 Induces Efficient Degradation of AGO Proteins

**Figure 2:**
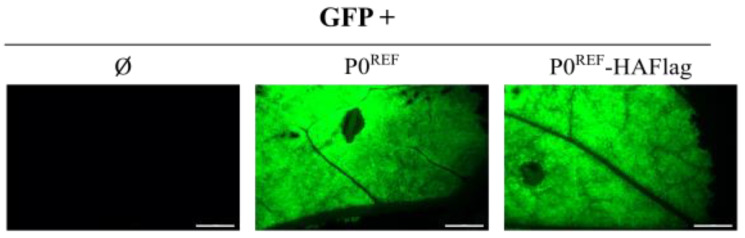
Corroboration of the preservation of VSR activity in a HAFlag-tagged PeVYV-5 P0^REF^. *N. benthamiana* leaves were agroinfiltrated with the construct for expression of GFP either alone (Ø) or in combination with constructs directing expression of PeVYV-5 P0^REF^ or PeVYV-5 P0^REF^-HAFlag. Pictures show GFP fluorescence in agroinfiltrated leaf patches at 10 dpif. The inset scale bar corresponds to 5 mm in all panels.

**Figure 3:**
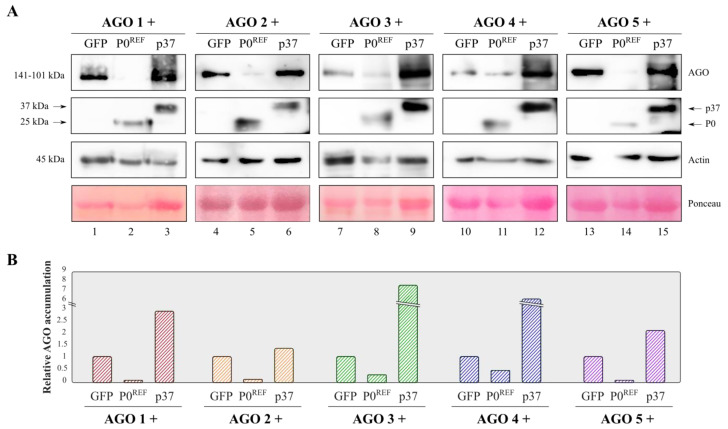
Analysis of accumulation levels of AGO proteins in the presence of PeVYV-5 P0^REF^. *N. benthamiana* leaves were agroinfiltrated with constructs for expression of HA-tagged AGO1, AGO2, AGO3, AGO4 or AGO5 in combination with constructs directing expression of GFP, PeVYV-5 P0^REF^-HAFlag or PLPV p37-HAFlag. At 3 dpif, protein extracts were prepared from agroinfiltrated leaves and subjected to Western blot analysis with an anti-HA antibody (**A**). Estimated protein sizes and positions of AGO, P0^REF^ and p37 tagged proteins are indicated at the left and right of autoradiograms, respectively. Ponceau S staining is shown below the blots as loading control. An anti-actin antibody was also employed both as additional loading control and to quantify relative accumulation levels of AGO proteins which are represented graphically in (**B**).

As mentioned above, results with several P0 proteins have led to the proposal that the VSR activity of these viral products relies on AGO degradation [20,21]. To investigate whether this was also the case with PeVYV-5 P0, the stability of distinct AGO proteins was assessed in the presence of this VSR. In order to track the expression of P0^REF^, this polypeptide was firstly fused to a composed HAFlag tag and the VSR activity of the tagged protein (P0^REF^-HAFlag) was corroborated through a suppression assay. The results showed that P0^REF^-HAFlag was able to inhibit RNA silencing as efficiently as the untagged P0^REF^, since both versions of the protein promoted high and comparable fluorescence levels when co-expressed with GFP (Figure 2). Next, *N. benthamiana* leaves were infiltrated with the P0^REF^-HAFlag construct in combination with constructs directing expression of distinct HA-tagged AGOs, more specifically AGO1, AGO2, AGO3, AGO4 and AGO5. The same AGO proteins were co-expressed with GFP, a non-VSR protein, or with p37 (tagged with HAFlag), a VSR which exerts its function through sRNA binding [9]. Protein extracts were prepared from the agroinfiltrated plant material and subjected to Western blot analyses with an anti-HA antibody. These analyses showed that accumulation levels of any AGO protein were severely reduced in tissues co-expressing P0^REF^ in comparison with those co-expressing the control GFP (Figure 3 and Figure S3). In contrast, the co-expression of p37 resulted in a remarkable increase in AGO contents as expected for a VSR which acts through sRNA sequestration. In short, the results indicated that P0 is able to induce decay of AGO1, one of the main effectors in antiviral defense, as well as of other AGO proteins. 

### 3.3. Analysis of Natural Variability of PeVYV-5 P0 Reveals Considerable Conservation

So far, sequence data on PeVYV-5 are limited to the isolate here called reference isolate which was identified in Almería (Spain) in 2013, when an important viral outbreak was reported [24,30]. In order to explore potential natural variability of PeVYV-5 P0, six pepper plants tested positive for the virus and collected in 2020 and 2021 in greenhouses of Almería region (see Material and Methods), were employed for RT-PCR amplification of the P0 gene. Sequencing of the PCR products confirmed the virus identity and the PeVYV-5 isolates were named as BN1-4 and VC1-2. Sequence comparisons showed considerable conservation of the P0 gene between isolates (nucleotide identities ranging from 98 to 100% in pairwise comparisons). Alignment of the nucleotide sequences showed 17 polymorphic positions (2.26% of the positions in the alignment) that were distributed along the coding region and that corresponded to nucleotide replacements (Supplementary Figure S4). Ten of these nucleotide replacements gave rise to changes at the amino-acid level. The ratio between non-synonymous (d_N_) and synonymous (d_S_) substitution rates was calculated as an indication of the direction and intensity of the selective constraints operating in the P0 coding region. The calculated dn was significantly smaller than ds (d_N_/d_S_ = 0.6956), supporting the notion that purifying selection has been acting as a major force for diversification of P0 between isolates [38]. 

**Figure 4:**
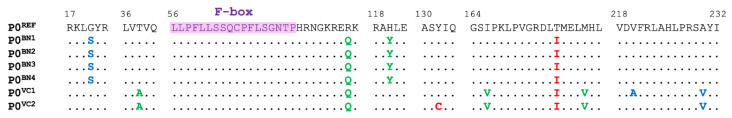
Partial alignment of P0 proteins encoded by distinct PeVYV-5 isolates. P0 sequence of the reference isolate [24] is depicted at the top of the alignment though only segments enclosing polymorphic residues are shown. Numbers above the reference sequence correspond to positions in the complete protein. Residues conserved in all isolates are indicated by dots. The F-box-like motif is displayed on a purple background, and non-conservative, semiconservative and conservative amino acid substitutions are shown with red, blue and green letters, respectively.

Consistent with the data at nucleotide level, sequence identities of P0 proteins encoded by the distinct isolates were high and ranged from 97 to 100%. As mentioned above, ten polymorphic positions out of the 249 positions in the alignment were detected, representing a percentage of 4.02% (Figure 4). The variable positions were located throughout the protein and only two amino acid replacements were non-conservative whereas the remaining ones were either conservative or semiconservative (Figure 4). None of the identified substitutions affected the F-box-like motif. In conclusion, the results corroborated, on one side, the presence of PeVYV-5 in recent campaigns in Spain and, on the other, indicated that the P0 protein of PeVYV-5 may be under negative selection though may tolerate some sequence variation. 

### 3.4. Alterations in Several Motifs of PeVYV-5 P0 Abolish Its VSR Function Concurrently with Its Capability to Induce AGO Degradation

**Figure 5:**
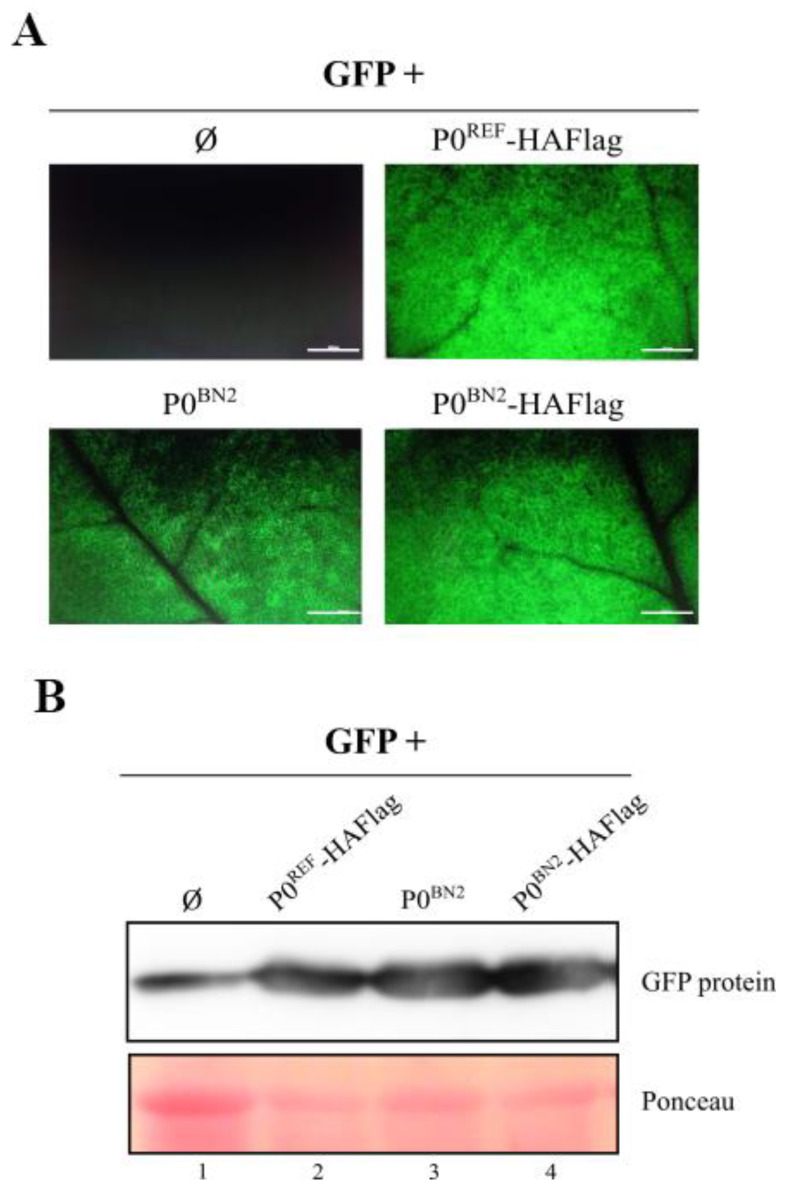
Evaluation of RNA silencing suppressor activity of the natural variant PeVYV-5 P0^BN2^. *N. benthamiana* leaves were agroinfiltrated with construct for expression of GFP either alone (Ø) or in combination with constructs directing expression of P0^REF^-HAFlag, P0^BN2^ or P0^BN2^-HAFlag. (**A**) GFP fluorescence at 10 dpif in infiltrated leaf patches. The inset scale bar corresponds to 5 mm in all panels. (**B**) Western blot for detection of GFP. Ponceau S staining is shown below Western results as loading control.

Some previous studies have shown significant differences in the VSR activity of proteins P0 encoded by isolates of a given polerovirus [42,43,44]. We selected protein P0 of one of the new identified PeVYV-5 isolates, BN2, to evaluate its capability for suppressing RNA silencing. Co-expression of P0^BN2^, either untagged or with a HAFlag tag, with GFP led to intense fluorescence at 10 dpif and beyond, which were indeed comparable to that observed with HAFlag-tagged P0^REF^ (Figure 5A). In contrast, GFP fluorescence was barely detectable in leaves agroinfiltrated with just the GFP construct at 10 dpif. A Western blot analysis of the protein extracts from the agroinfiltrated tissues revealed a correspondence between fluorescence and GFP accumulation levels (Figure 5B and Figure S5). The results indicated that PeVYV-5 P0 may accommodate some degree of sequence polymorphism without negatively impacting its VSR function. 

**Figure 6:**
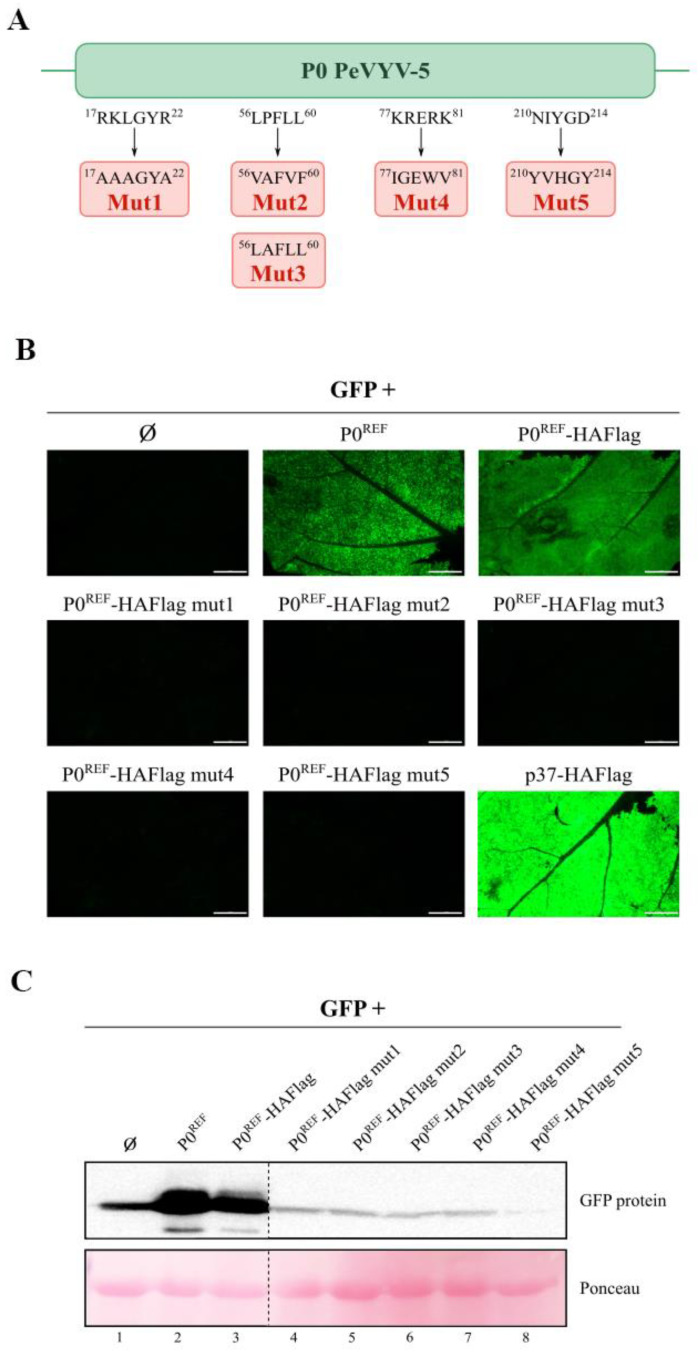
Analysis of RNA silencing suppressor activity of engineered variants of PeVYV-5 P0^REF^. Distinct nucleotide substitutions were engineered through site-directed mutagenesis into (HAFlag-tagged) P0^REF^ gene giving rise to amino acid replacements in the derived proteins. The mutated constructs (mut1–5) were co-infiltrated with the GFP construct in *N. benthamiana* leaves. The GFP construct was also agroinfiltrated alone (Ø) or in combination with the construct directing expression of HAFlag-tagged P0^REF^, to be used as control samples. (**A**) Schematic representation of the P0^REF^ protein with the amino acid replacements present in each engineered variant. (**B**) GFP fluorescence at 10 dpif in infiltrated leaf patches. The inset scale bar corresponds to 5 mm in all panels. (**C**) Western blot analysis for detection of GFP. Ponceau S staining is shown below Western results as loading control.

In the view of the above results, we decided to alter some motifs in the HAFlag-tagged P0^REF^ protein to assess their relevance for VSR activity. Five mutant versions of the protein were generated (Figure 6A) and subjected to an RNA silencing assay. The results revealed that all the engineered sets of mutations abolished VSR function, supporting the contribution of distinct parts of the protein to such crucial role (Figure 6B,C and  Figure S6). 

**Figure 7:**
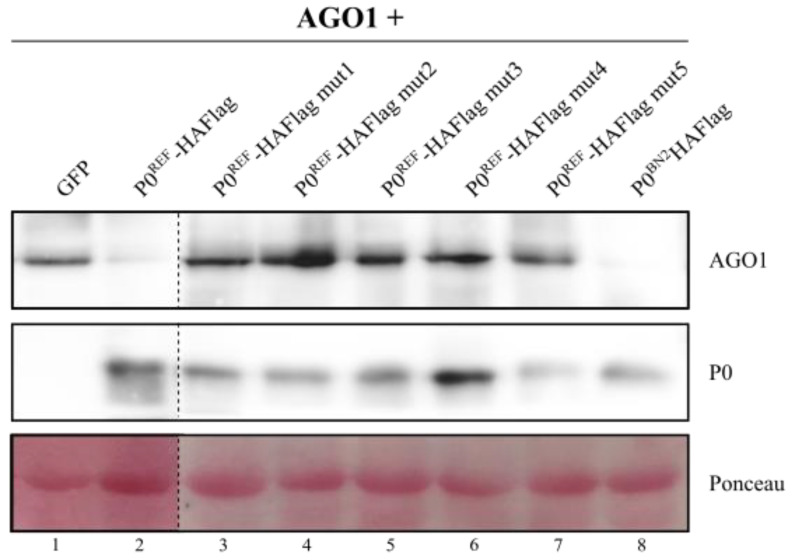
Assessment of accumulation levels of AGO1 in the presence of natural and engineered PeVYV-5 P0 variants. *N. benthamiana* leaves were agroinfiltrated with constructs for expression of HA-tagged AGO1 in combination with constructs directing expression of GFP, P0^REF^-HAFlag, P0^REF^-HAFlag mut1 to 5, or P0^BN2^-HAFlag. At 3 dpif, protein extracts were prepared from agroinfiltrated leaves and subjected to Western blot analysis with an anti-HA antibody which allowed detection of AGO1 (upper blot) and of the distinct P0 variants (lower blot). Ponceau S staining is shown below the blots as loading control.

Finally, we evaluated the capacity of the distinct (natural or artificial) P0 variants to promote degradation of AGO proteins. We employed AGO1 for this purpose together with the different HAFlag-tagged P0 proteins or with GFP as a non-VSR protein. The results showed that, as seen above (Figure 3), P0^REF^ induced a decrease in AGO1 accumulation, and the same was observed with P0^BN2^ (Figure 7, upper blot, and Supplementary Figure S7). In contrast, mutants 1 to 5 of P0^REF^ did not have such an influence on AGO1 levels that remained comparable to those found in plant patches co-expressing GFP and AGO1. The observed effects were not related to differences in the stability of P0 variants as all of them were detected at similar levels (Figure 7, lower blot, and Supplementary Figure S7). Altogether, the results supported a positive correlation between the VSR function of PeVYV-5 P0 and its ability to elicit AGO degradation. 

## 4. Discussion

VSRs are critical viral factors for virus viability and, thus, appealing targets for virus control. In this work, we have pursued to evaluate the putative VSR activity of protein P0 encoded by PeVYV-5, an emerging pathogen that jeopardizes pepper crops. The results have allowed us to corroborate the capability of the protein to block RNA silencing, which is comparable in strength and persistency to that of potent VSRs such as PLPV p37 [9]. Analyses of AGO accumulation in the presence of PeVYV-5 P0 have revealed that this VSR induces AGO decay, in line with that reported for a series of P0 proteins [20,21,23,43,45,46]. The induced instability was not restricted to AGO1, one of the major antiviral AGOs, but to all the assayed AGOs (AGO1-5). A similar situation was reported for P0 products encoded by *Beet western yellows virus* and *Turnip yellows virus* [20,47], suggesting that P0 proteins are able to elicit clearance of any AGO component, most likely because of the presence of common degron(s) in these endonucleases, as supported by recent studies [23]. In any case, though AGO1 has been noted as a chief actor in antiviral silencing, other AGOs significantly contribute to the antiviral response [9,13,22], and indiscriminate AGO targeting by P0 may well be advantageous for the success of viral infection.

As mentioned previously, molecular description of PeVYV-5 was limited so far to one viral isolate collected in 2013 in Almería (Spain) [24,30]. Here, we have confirmed the presence of the virus in pepper samples gathered in 2020–2021 in the same region, indicating that this pathogen continues to circulate and that the risk of new viral outbreaks cannot be dismissed. Focusing the attention on P0 gene and protein, limited heterogeneity was detected at nucleotide and amino acid levels and the data supported that the protein is under negative selection. Studies with other poleroviruses have revealed remarkable differences in the VSR activity of P0 proteins encoded by distinct isolates, even when the corresponding sequences had scarce dissimilarities [43,44]. The analysis of the VSR function of a natural variant of PeVYV-5 P0, P0^BN2^, differing in four positions with regard the protein of the reference isolate (Figure 4), has shown strict maintenance of the suppressor capability. Despite these results indicated that the protein may accommodate sequence heterogeneity at certain positions without losing function, assays with engineered P0 mutants have revealed that this is not the case for some motifs of the protein. Indeed, the F-box-like motif has proven to be critical for VSR activity of PeVYV-5 P0, with the mutation of a single landmark residue (mutant 3) being sufficient to completely abolish the performance of the protein (Figure 6). Two stretches rich in basic amino acids and a segment near the C-terminus have likewise been revealed as essential for VSR function. The relevance of the F-box-like motif has been previously highlighted for some P0 proteins, though the specific role it plays is still unclear [46,47,48]. Determining the molecular consequences that mutations in this region and others of PeVYV-5 P0 may have will require further research.

Finally, a strict correlation between the VSR activity of PeVYV-5 P0 and the capability of the protein to promote AGO degradation has been found, highly supporting that the former relies on the latter, in line with that proposed for P0 encoded by some poleroviruses [45,46]. Previous studies have supported the involvement of the autophagy pathway (*versus* the 26S proteasome pathway) in the AGO decay elicited by, at least, the few P0 proteins for which this question has been investigated that, more specifically, corresponded to the VSRs encoded by *Beet western yellows virus* [47], *Brassica yellows virus* [45] and *Turnip yellows virus* [49]. This finding marks a difference from protein p25, the VSR encoded by *Potato virus* X (genus *Potexvirus*), which has been reported to induce proteasomal degradation of AGO1 [14]. Experiments are under way to ascertain which proteolytic machinery mediates AGO degradation induced by PeVYV-5 P0 and, more intriguingly, which specific host components are involved.

## 5. Conclusions

In summary, in this work we have proven VSR activity of the P0 protein encoded by PeVYV-5, a pathogen closely related to other emerging poleroviruses threatening peppers in distinct parts of the world. High mutation and recombination rates seem to contribute to the rapid diversification of this group of viruses [19,24,50], which may lead to important shifts in molecular and biological properties, as illustrated by recent reports [50]. Hence, in order to develop control measures to prevent extensive damages, it seems essential both to deepen the knowledge and to track the occurrences of these infectious agents. In this sense, we have provided data showing that PeVYV-5 is still present in Spain and that the risk of new outbreaks cannot be ruled out.

We have also gained insights into motifs/segments contributing to the VSR function of PeVYV-5 P0 through the analysis of natural and engineered protein variants. The results have, moreover, supported that such a function relies on the removal of many, if not all, AGO proteins in a likely attempt of the virus to leave the host helpless against infection. Further studies may shed light on the precise timing of the distinct events, their cellular compartmentalization and the host machinery involved. 

## Figures and Tables

**Table 1:** List of primers used in this work.

Primer	Position ^a^	Sequence ^b^	Constructs/ RT-PCR
CH857 CH857	51-72 (S) 779-801 (AS)	5′-GTGGATCCATGAACTTTGAATTGATCAAC-3′ (*Bam*HI) 5′-GACTGCAGTCACTGTAGTTCCTCCTGAATC-3′ (*Pst*I)	35S:P0^REF^ 35S:P0^BN2^
CH857 CH868	51-72 (S) 779-798 (AS)	5′-GTGGATCCATGAACTTTGAATTGATCAAC-3′ (*Bam*HI) 5′-GGCTGCAGAGCGTAATCTGGAACATCGTATGGGTACTGTAGTTCCTCCTGAATC-3′ (*Pst*I)	35S:P0^REF^-HAFlag 35S:P0^BN2^-HAFlag
CH882P CH883P	81-136 (S) 81-136 (AS)	5′-CTGAAAGTTTCCCTCACTGCCGCGGCCGGTTACGCAGAGAGAATCCTAAATTTAG-3′ 5′-CTAAATTTAGGATTCTCTCTGCGTAACCGGCCGCGGCAGTGAGGGAAACTTTCAG-3′	35S:P0^REF^-HAFlag mut1
CH884P CH885	200-245 (S) 200-245 (AS)	5′-CTCTATTTGTGCTCTCGTCGCTTTCGTTTTCAGCAGCCAATGTCC-3′ 5′-GGACATTGGCTGCTGAAAACGAAAGCGACGAGAGCACAAATAGAG-3′	35S:P0^REF^-HAFlag mut2
CH886 CH887	200-245 (S) 200-245 (AS)	5′-CTCTATTTGTGCTCTCCTCGCTTTCCTTCTCAGCAGCCAATGTCC-3′ 5′-GGACATTGGCTGCTGAGAAGGAAAGCGAGGAGAGCACAAATAGAG-3′	35S:P0^REF^-HAFlag mut3
CH888 CH889	264-313 (S) 264-313 (AS)	5′-CCGCACCGGAACGGCATCGGGGAATGGGTCCGAGTCTCTAAGCTCGCTC-3′ 5′-GAGCGAGCTTAGAGACTCGGACCCATTCCCCGATGCCGTTCCGGTGCGG-3′	35S:P0^REF^-HAFlag mut4
CH890 CH891	662-707 (S) 662-707 (AS)	5′-TGCTTTATGCCTTCACTACGTTCATGGTTATGGTA TTGCTGTGGA-3′ 5′-TCCACAGCAATACCATAACCATGAACGTAGTGA AGGCATAAAGCA-3′	35S:P0^REF^-HAFlag mut5
CH911 CH912	1-31 (S) 808-832 (AS)	5′-CGTCTAGACAAAATATACGAAGAGAGAGAGCCCTTGC-3′ 5′-GCCTGCAGCAAAGAAATAAATCCCTTAACTTG-3′	RT-PCR

^a^ Positions covered by the primers in the PeVYV-5 genome (Genbank number accession: KY523072.1). (S) and (AS): sense and antisense. ^b^ Engineered restriction sites for cloning purposes are underlined. HA tag is shown in blue and mutations are highlighted in red.

## Data Availability

The data presented in this study are available in [insert article or supplementary material here].

## Supplementary Materials

The following supporting information can be downloaded at: https://www.mdpi.com/article/10.3390/biology11121801/s1, Figure S1: Assessment of RNA silencing suppressor activity of PeVYV-5 P0^REF^, Figure S2: Western blot for detection of GFP, Figure S3: Western blot for detection of AGO proteins, PeVYV-5 P0, PLPV p37 and actin, Figure S4: Analysis of PeVYV-5 P0 gene variability, Figure S5: Western blot for detection of GFP, Figure S6: Western blot for detection of GFP, Figure S7: Western blot for detection of AGO1 and wt and mutant PeVYV-5 P0 proteins.
