# Supplementary material for: Assessment of the RNA Silencing Suppressor Activity of Protein P0 of Pepper Vein Yellows Virus 5: Uncovering Natural Variability, Relevant Motifs and Underlying Mechanism
Source: Biology (Basel). 2022 Dec 12;11(12):1801. doi: 10.3390/biology11121801 (PMC9775047; doi:10.3390/biology11121801)
Supplement: Supplementary file 1 [file biology-11-01801-s001.zip › biology-2059097-supplementary.pdf]

SUPPLEMENTARY FIGURES

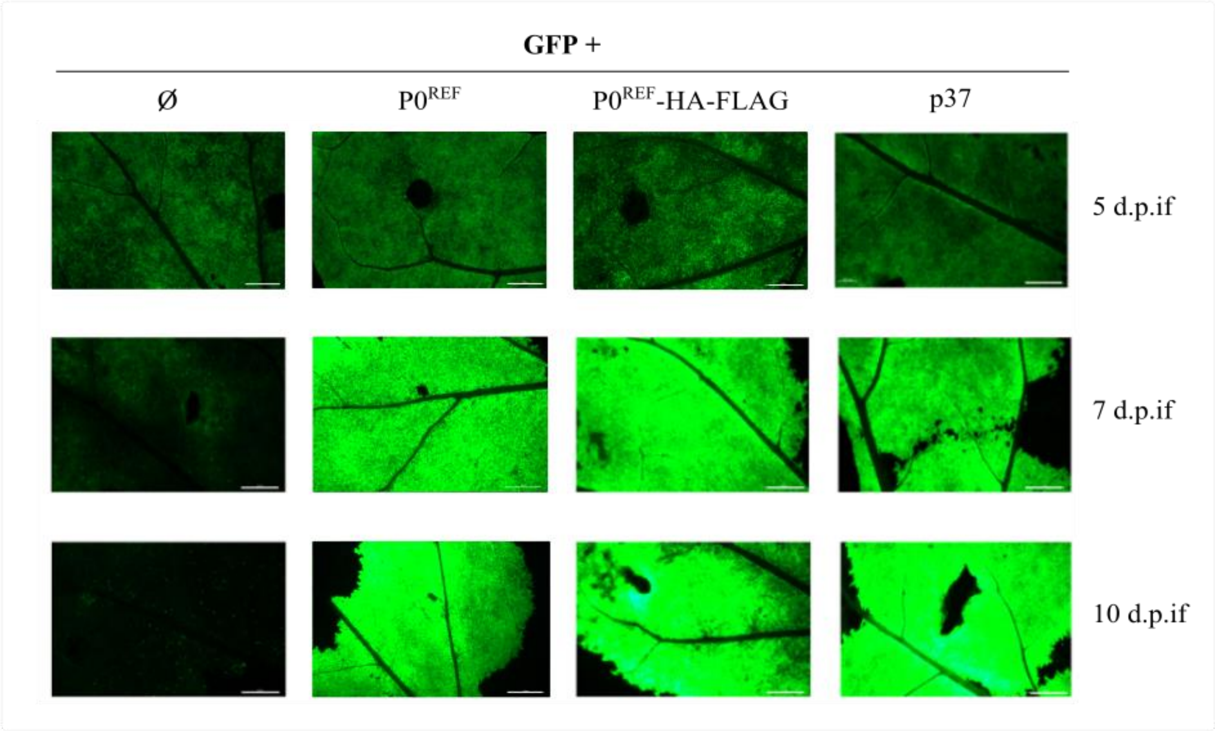

Figure S1. Assessment of RNA silencing suppressor activity of PeVYV-5 P0REF. *N. benthamiana* leaves were agroinfiltrated with constructs for expression of GFP either alone (Ø) or in combination with constructs directing expression of PeVYV-5 P0REF or PLPV p37. GFP fluorescence at 5, 7 and 10 d.p.if. in infiltrated leaf patches. The inset scale bar corresponds to 5 mm in all panels.

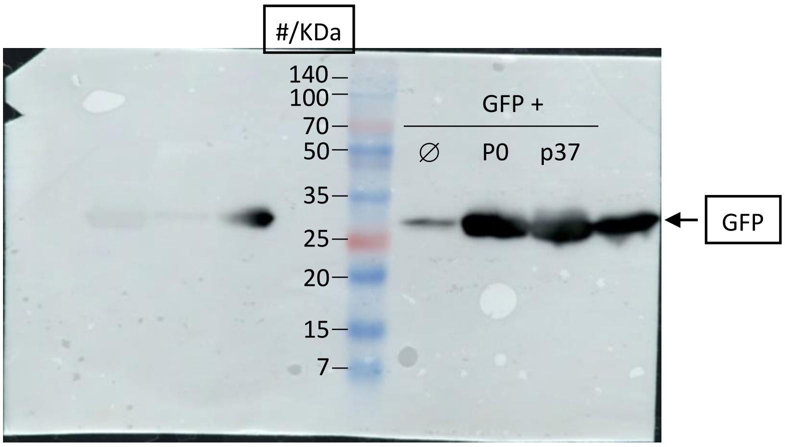

Figure S2. Western blot for detection of GFP. Proteins separated in polyacrylamide gel (12 %) were transferred to polyvinylidene difluoride (PVDF) membranes (Roche Diagnostics GmbH) by semidry electroblotting (1 mA per cm^2, 1 h). Membrane was incubated with anti-GFP (11 814 460 001, 1:1000, Roche Diagnostics GmbH) antibody for detection of GFP (~27 kDa) protein, later on with a sheep anti-mouse horseradish peroxidase-conjugated secondary antibody (NA931, 1:10000, GE Healthcare) and signals were developed with Western Blot Ultra Sensitive HPR substrate (#T7104A, Takara Bio INC). #Weight marker (molecular weight in kDa): Perfect Color Protein Ladder, 7 to 240 kDa; catalogue number: E3215. Signals were recorded with the aid of an

Amersham ImageQuant™ 800 GxP biomolecular imager (Cytiva). Different images can be obtained with ImageQuant. Here, chemiluminescence with marker is shown but, for the figure, we used an image only with chemiluminescence signal and we adjust the color levels with Gimp (GIMP 2.10, The GIMP Development Team, retrieved from https://www.gimp.org).

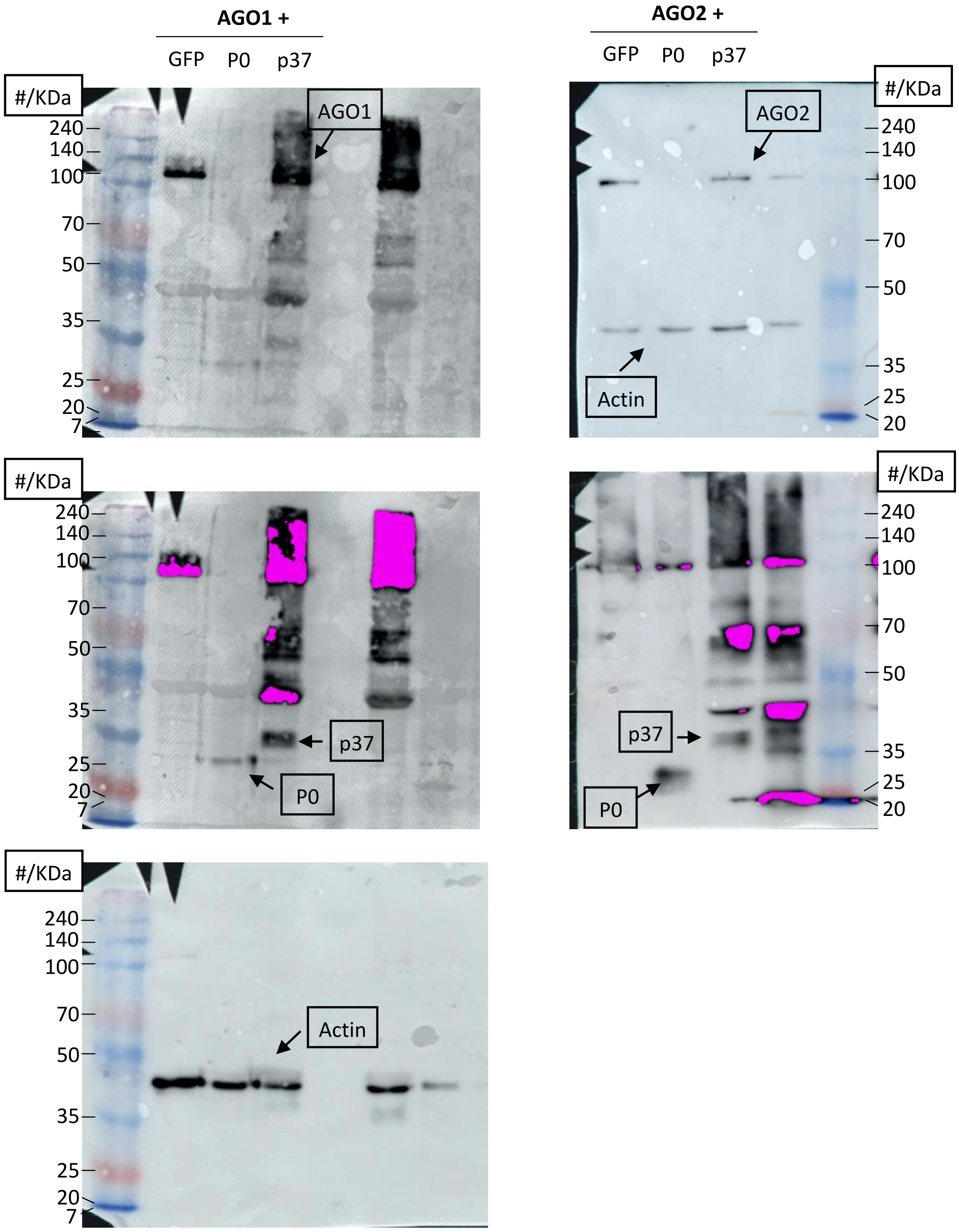

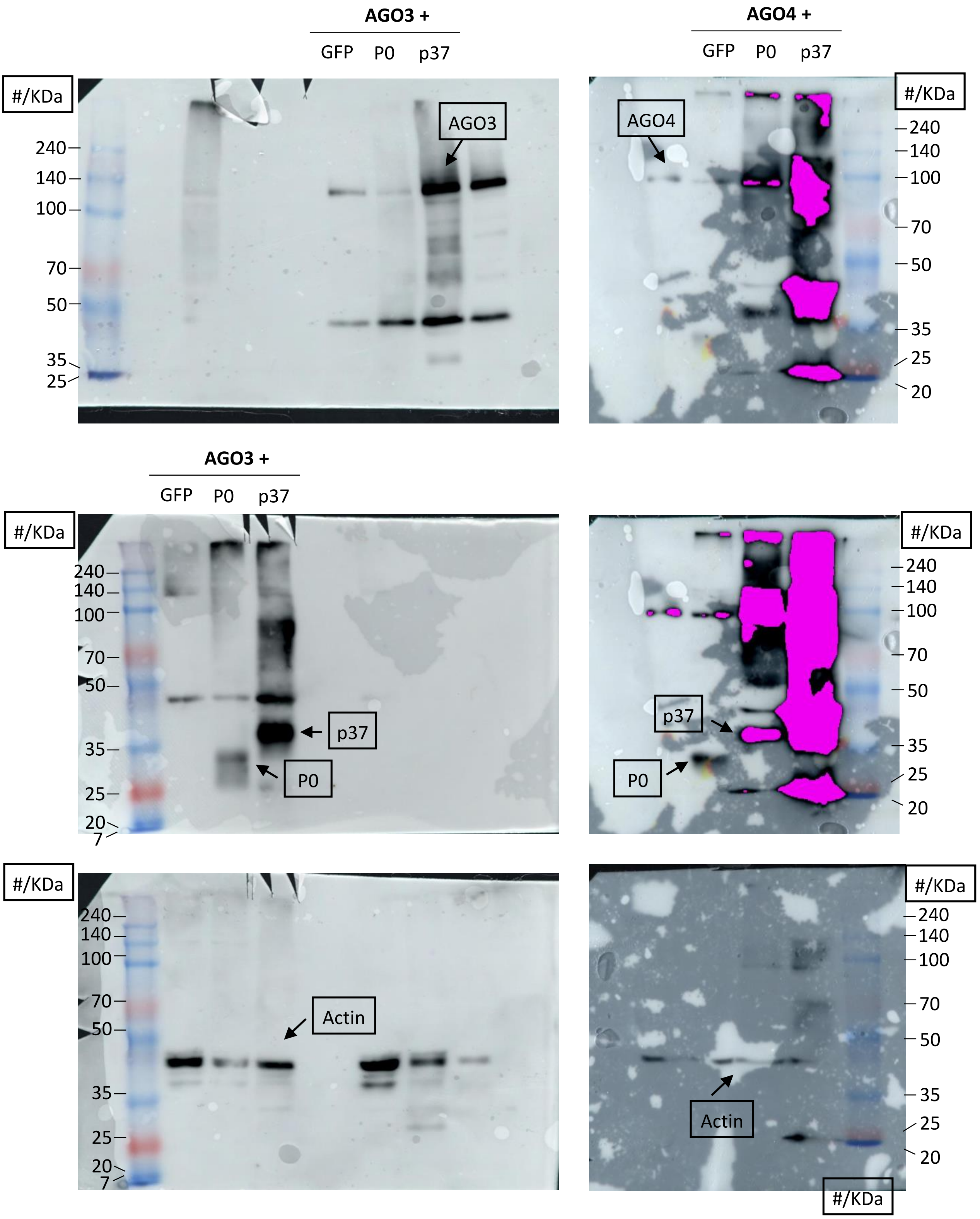
AGO3 +
GFP
P0
p37
AGO4 +
GFP
P0
p37
#/KDa
AGO3
240
140
100
70
50
35
25
AGO4
20
AGO3 +
GFP
P0
p37
p37
P0
7
Actin
#/KDa

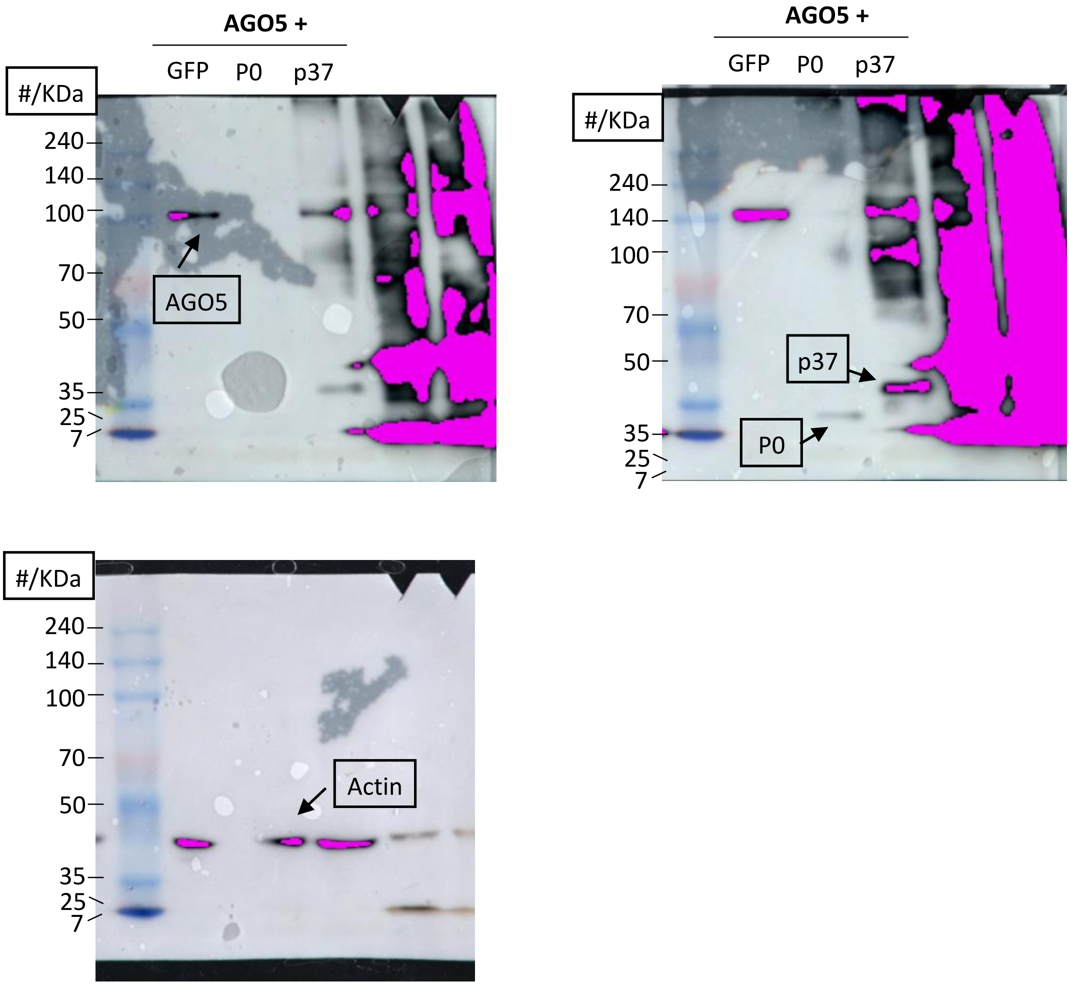

Figure S3. Western blot for detection of AGO proteins, PeVYV-5 P0, PLPV p37 and actin. Proteins separated in polyacrylamide gels (8 %) were transferred to polyvinylidene difluoride (PVDF) membranes (Roche Diagnostics GmbH) by semidry electroblotting (1 mA per cm^2, 1 h). Membranes were incubated with an anti-HA horseradish peroxidase-conjugated primary antibody (3F10; 1:3000; Roche Diagnostics GmbH) for detection AGO proteins (~101-141 kDa), P0 (~29 kDa), and p37 (~37 kDa), and signals were developed with Western Blot Ultra Sensitive HPR substrate (#T7104A, Takara Bio INC). Actin (~37 kDa) was detected with a rabbit anti-actin antibody (AS13 2640; 1:5000; Agrisera) and a goat anti-rabbit horseradish peroxidase-conjugated secondary antibody (AS09 602; 1:10000; Agrisera). # Weight marker (molecular weight in kDa): Perfect Color Protein Ladder, 7 to 240 kDa; catalogue number: E3215. Signals were recorded with the aid of an Amersham ImageQuant™ 800 GxP biomolecular imager (Cytiva). For AGO3, different gels were run with the same samples to detect all proteins. Different exposures of chemiluminescence with marker are shown here. Some of them show saturation in pink, but we selected these exposures just to show good signals of the different proteins, although these were not used to quantify and normalize the data. For the final Figure, we used images only with chemiluminescence signals and we adjusted the color levels with Gimp (GIMP 2.10, The GIMP Development Team, retrieved from https://www.gimp.org).

a)

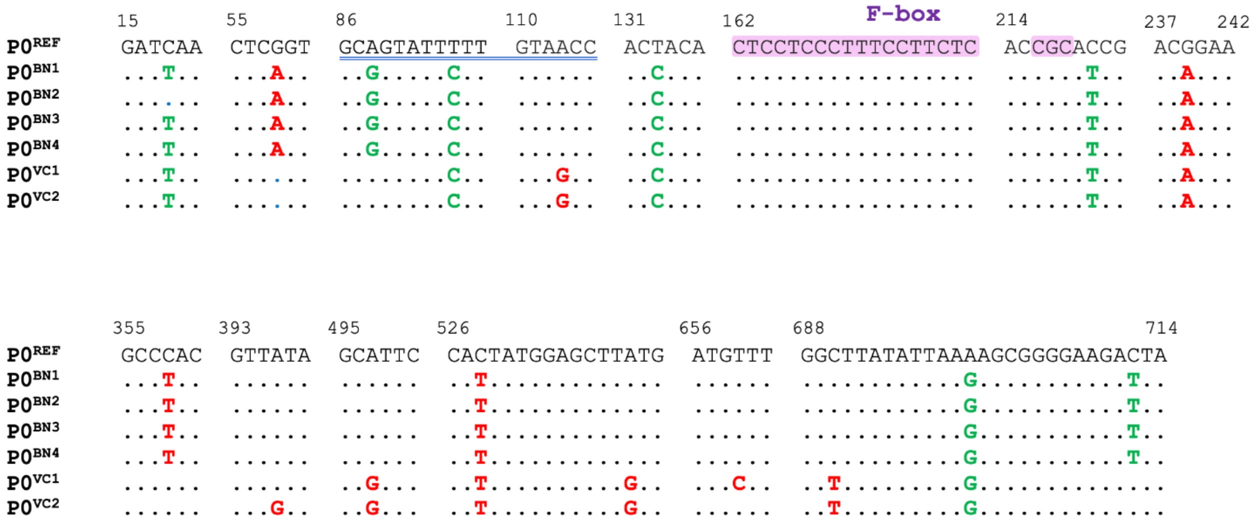
F-box
15 55 86 110 131 162 214 237 242
P0REF GATCAA CTCGGT GCAGTATTTTT GTAACC ACTACA CTCCTCCCTTTCCTTCTC ACCGCACCG ACGGAA
P0BN1
P0BN2
P0BN3
P0BN4
P0VC1
P0VC2
355 393 495 526 656 688 714
P0REF GCCCAC GTTATA GCATTC CACTATGGAGCTTATG ATGTTT GGCTTATATTAAAAGCGGGGAAGACTA

(b)

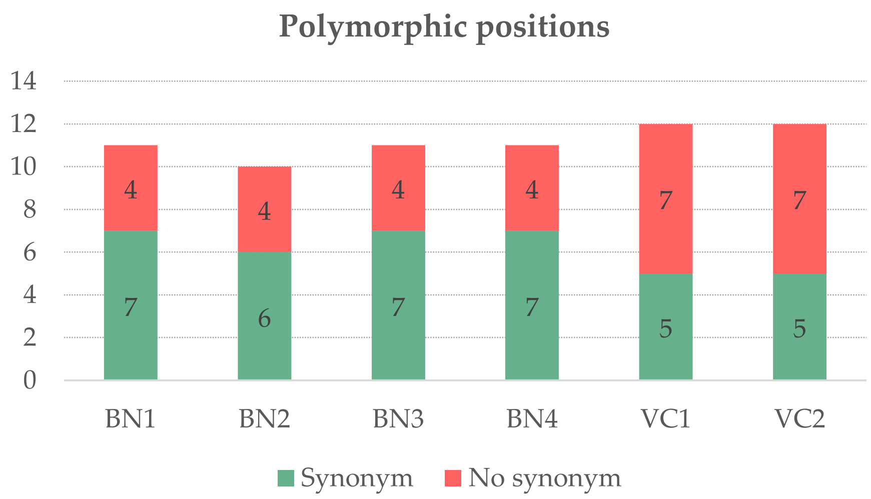
Polymorphic positions
14
12
10
8
6
4
2
0
BN1
BN2
BN3
BN4
VC1
VC2
Synonym
No synonym

(c)

	REF	BN1	BN2	BN3	BN4	VC1	VC2
**REF**	-	739/750 (99%)	740/750 (99%)	739/750 (99%)	739/750 (99%)	738/750 (98%)	738/750 (98%)
**BN1**	245/249 (98%)	-	749/750 (99%)	750/750 (100%)	750/750 (100%)	741/750 (99%)	741/750 (99%)
**BN2**	245/249 (98%)	249/249 (100%)	-	749/750 (99%)	749/750 (99%)	740/750 (99%)	740/750 (99%)
**BN3**	245/249 (98%)	249/249 (100%)	249/249 (100%)	-	750/750 (100%)	741/750 (99%)	741/750 (99%)
**BN4**	245/249 (98%)	249/249 (100%)	249/249 (100%)	249/249 (100%)	-	741/750 (99%)	741/750 (99%)
**VC1**	242/249 (97%)	242/249 (97%)	242/249 (97%)	242/249 (97%)	242/249 (97%)	-	748/750 (99%)
**VC2**	242/249 (97%)	242/249 (97%)	242/249 (97%)	242/249 (97%)	242/249 (97%)	247/249 (99%)	-

Figure S4. Analysis of PeVYV P0 gene variability. (a) Alignment of P0 nucleotidic sequences of the distinct isolates reported in the study. Synonym changes are shown in green, non-synonym positions in red and F-box domain in yellow. (b) Graph showing the synonym and non-synonym polymorphic positions for each isolate. (c) Sequence identities of P0 proteins. Identities of nucleotide sequences are shown in orange and protein sequence identities in blue.

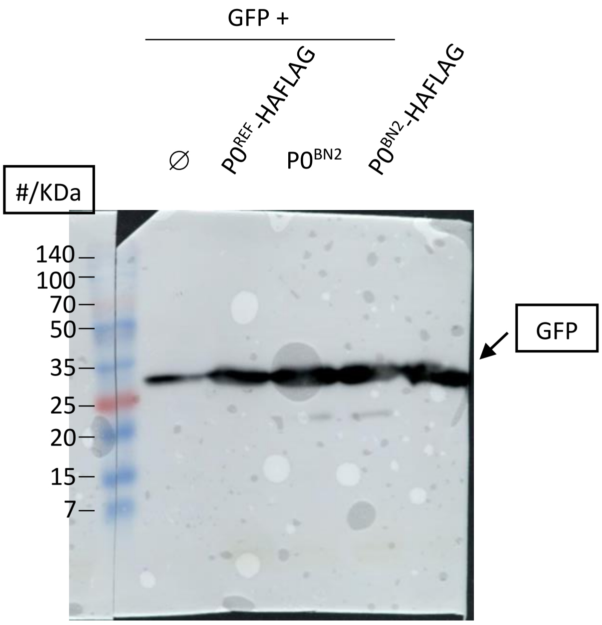

Figure S5. Western blot for detection of GFP. Proteins separated in polyacrylamide gel (12 %) were transferred to polyvinylidene difluoride (PVDF) membranes (Roche Diagnostics GmbH) by semidry electroblotting (1 mA per cm^2, 1 h). Membranes were incubated with anti-GFP (11 814 460 001, 1:1000, Roche Diagnostics GmbH) antibody for GFP (~27 kDa) detection, later on with a sheep anti-mouse horseradish peroxidase-conjugated secondary antibody (NA931, 1:10000, GE Healthcare), and signals were developed with Western Blot Ultra Sensitive HPR substrate (#T7104A, Takara Bio INC). #Weight marker (molecular weight in kDa): Perfect Color Protein Ladder, 7 to 240 kDa; catalogue number: E3215. Signals were recorded with the aid of an Amersham ImageQuant™ 800 GxP biomolecular imager (Cytiva). Different images can be obtained with ImageQuant. Here, chemiluminescence with marker is shown but, for the figure, we use an image only with chemiluminescence signal and we adjust the color levels with Gimp (GIMP 2.10, The GIMP Development Team, retrieved from https://www.gimp.org).

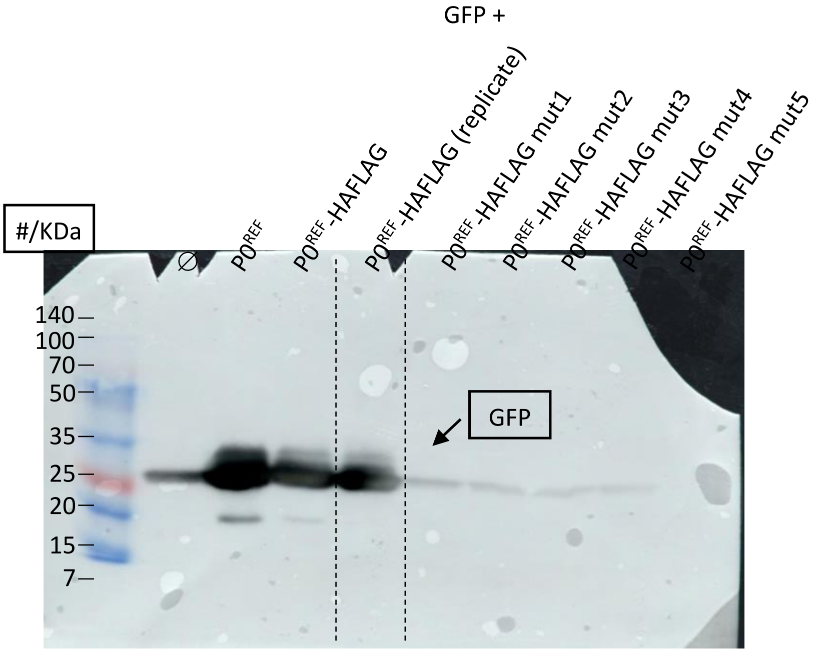

Figure S6. Western blot for detection of GFP. Proteins separated in polyacrylamide gel (12 %) were transferred to polyvinylidene difluoride (PVDF) membranes (Roche Diagnostics GmbH) by semidry electroblotting (1 mA per cm^2, 1 h). Membranes were incubated with anti-GFP (11 814 460 001, 1:1000, Roche Diagnostics GmbH) antibody for GFP (~27 kDa) detection, later on with a sheep anti-mouse horseradish peroxidase-conjugated secondary antibody (NA931, 1:10000, GE Healthcare), and signals were developed with Western Blot Ultra Sensitive HPR substrate (#T7104A, Takara Bio INC). #Weight marker (molecular weight in kDa): Perfect Color Protein Ladder, 7 to 240 kDa; catalogue number: E3215. Signals were recorded with the aid of an Amersham ImageQuant™ 800 GxP biomolecular imager (Cytiva). Different images can be obtained with ImageQuant. Here, chemiluminescence with marker is shown but, for the figure, we use an image only with chemiluminescence signal and we adjust the color levels with Gimp (GIMP 2.10, The GIMP Development Team, retrieved from https://www.gimp.org).

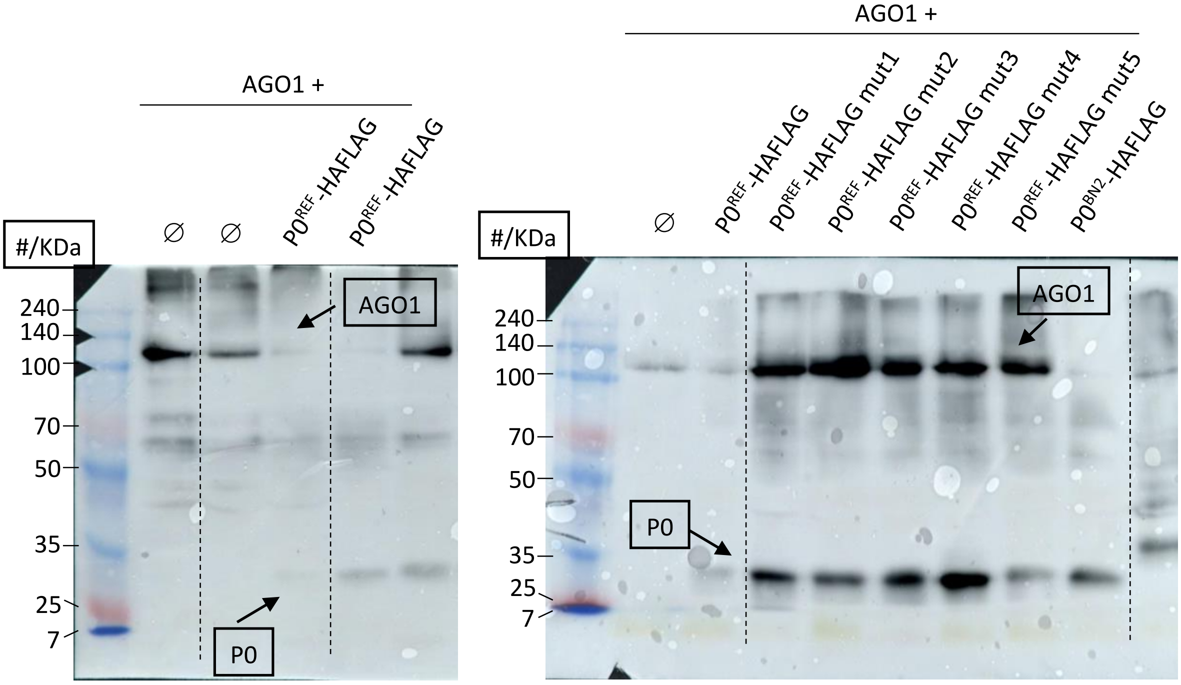

Figure S7. Western blot for detection of AGO1 and wt and mutant PeVYV-5 P0 proteins. Proteins separated in polyacrylamide gels (8 %) were transferred to polyvinylidene difluoride (PVDF) membranes (Roche Diagnostics GmbH) by semidry electroblotting (1 mA per cm^2, 1 h). Membranes were incubated with an anti-HA horseradish peroxidase-conjugated primary antibody (3F10; 1:3000; Roche Diagnostics GmbH) for AGO1 (~117 kDa) and P0 (~29 kDa) detection, and signals were developed with Western Blot Ultra Sensitive HPR substrate (#T7104A, Takara Bio INC). #Weight marker (molecular weight in kDa): Perfect Color Protein Ladder, 7 to 240 kDa; catalogue number: E3215. Signals were recorded with the aid of an Amersham ImageQuant™ 800 GxP biomolecular imager (Cytiva). Different images can be obtained with ImageQuant. Here, chemiluminescence with marker images are shown but, for the figure, we used images only with chemiluminescence signal and we adjust the color levels with Gimp (GIMP 2.10, The GIMP Development Team, retrieved from https://www.gimp.org).
